# Breakdown of antimicrobial host defense barrier as trigger of chronic inflammatory bowel diseases

**DOI:** 10.1186/1479-5876-9-S2-I14

**Published:** 2011-11-23

**Authors:** Jan Wehkamp

**Affiliations:** 1 Dr. Margarete Fischer-Bosch - Institute of Clinical Pharmacology, Internal Medicine I, Robert Bosch Hospital, Stuttgart, Germany

## 

Inflammatory bowel diseases are characterized by chronic intestinal inflammation at different sites. Data from animal models as well as human patients including gene-association studies suggest that different components of the innate barrier function are primarily defective. These recent advances support the evolving hypothesis that intestinal bacteria induce inflammation predominantly as a result of a weakened innate mucosal barrier in genetically predisposed individuals. The mechanisms include different defects in epithelial stem cell differentiation, defective pattern recognition, autophagy, endosomal stress and others. Secretory cells like the Paneth cell of the small intestinal crypt as well as mucin producing goblet cells seem to be of major importance in the initial stages of the disease process. This talk will discus our current understanding of the primary events of disease and also outline consequences for future therapeutic consequences. In summary, there is a need for therapeutic avenues aimed at restoring antimicrobial barrier function to prevent a bacterial-triggered inflammatory response.

